# Antimicrobial stewardship in Africa: A policy analysis of national action plans across five African countries

**DOI:** 10.1017/ash.2026.10303

**Published:** 2026-04-06

**Authors:** Hafeez Hamza, Samuel Olayinka Obafemi, Sylivia Juliet Nalugya, Gabriel Aseka, Lawan Abdulrazaq Ali, Paul M. Iziomo, Ayodele O. Majekodunmi, Estelle Mbadiwe

**Affiliations:** 1 Ducit Blue Foundation, Abuja, Federal Capital Territory, Nigeria; 2 Kwara State University, Nigeria; 3 Joint Clinical Research Centre, Uganda; 4 Bayero University, Nigeria; 5 Ajisefini Consulting, Abuja, Federal Capital Territory, Nigeria

## Abstract

Antimicrobial stewardship (AMS) is central to antimicrobial resistance (AMR) control, yet its prioritization varies across Africa. We analyzed AMS components of five African National Action Plans on AMR against global and regional policy benchmarks, identifying areas of alignment and critical gaps that should inform future AMR strategies.

## Introduction

Antimicrobial resistance (AMR) is a major and growing public health threat.^1^ Africa bears a disproportionate share of its burden,^1^ driven by high infectious disease prevalence, weak health systems, and widespread misuse and overuse of antimicrobials.^2^ Antimicrobial stewardship (AMS)—defined as coordinated interventions to optimize antimicrobial use—is a cornerstone of AMR control. Since 2015, countries have been encouraged to embed AMS within National Action Plans (NAPs) aligned with the World Health Organization (WHO) Global Action Plan (GAP), while African countries are additionally guided by the African Union (AU) Framework for AMR Control and, more recently, the AU AMR Landmark Report.^2–4^

The WHO Global Action Plan on AMR, adopted in 2015, provides the overarching framework guiding national and international responses to AMR through a multisectoral One Health (OH) approach.^3^ It outlines five strategic objectives, with optimization of antimicrobial use forming the core stewardship pillar against which national action plans are expected to align.

In the African context, the AU Framework for AMR Control (2020–2025) translates global AMR priorities into regionally relevant actions, reflecting the continent’s health system constraints and multisectoral realities.^4^ The framework emphasizes stewardship through actions listed under objectives two (delay emergence of AMR) and 4 (limit transmission of AMR).

The AU AMR Landmark Report (2024) articulates Africa’s strategic priorities for the next phase of AMR control, adopting a systems-thinking approach that foregrounds governance, political commitment, and sustainable health system strengthening.^2^ Stewardship is embedded within broader recommendations on access to quality medicines, workforce development, and coordinated continental action.

Together, these instruments represent the key global and regional reference points shaping national AMS strategies in Africa and provide the policy benchmarks used in this analysis. Given the widespread over-the-counter sales of prescription antimicrobials in Africa, AMS is critical for controlling AMR in the region. However, it is prioritized differently across settings. Therefore, this study assessed the alignment of AMS actions in NAPs of five African countries against the global and regional recommendations to better understand how African countries are translating stewardship principles into national AMR strategies. This study is based on intern projects from the One Health Pan-African AMR Internship/Mentorship Programme which is a capacity building and succession planning initiative of Ducit Blue Foundation.

## Method

**Figure 1.**
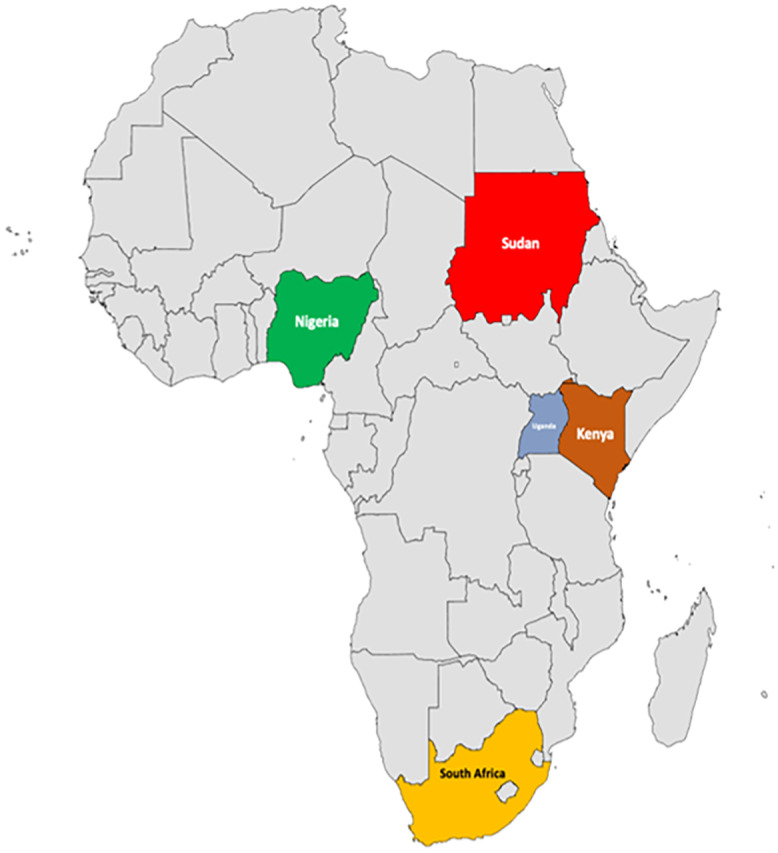
Spotlight Countries.

This study was conceived during the third cohort (2023–24) of the Ducit Blue Foundation’s (DBF) award-winning One Health Pan-African Internship/Mentorship Program, in partnership with One Health Lessons and the Nigerian Institute of Medical Research. A policy analysis was conducted to examine the AMS components of AMR NAPs of Kenya, Nigeria, South Africa, Sudan, and Uganda (Figure 1) against the WHO GAP, AU Framework on AMR Control 2020–2025, and AU AMR Landmark Report. The NAPs were obtained from the WHO Library of AMR NAPs.^5^ Each NAP was assessed against AMS actions in the GAP, the AU Framework on AMR Control, and the AU AMR Landmark Report, using a 3-point traffic light scale and Boolean operators to determine alignment.^6^

### Key findings

#### Alignment with the WHO global action plan

**Figure 2.**
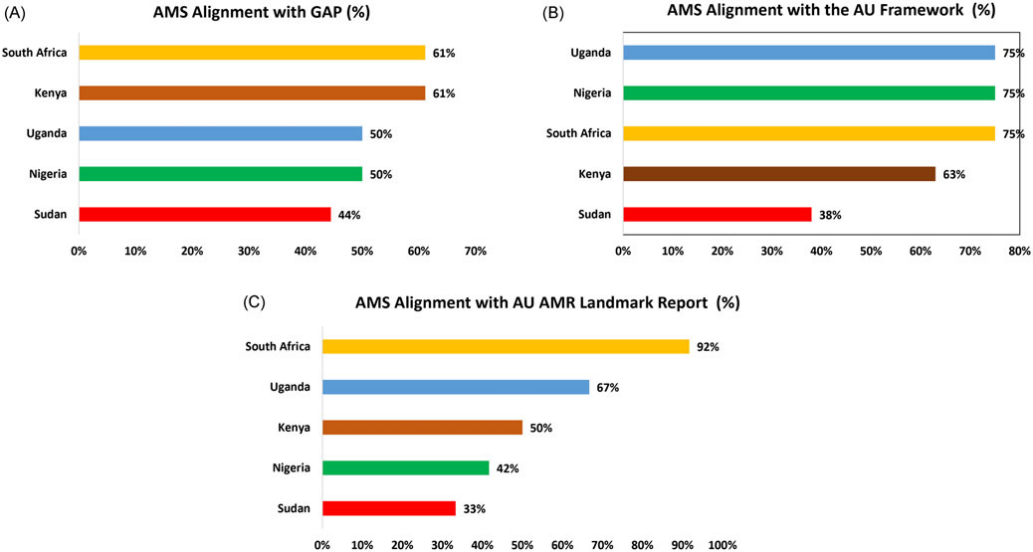
AMS alignment between selected NAPs and the GAP, AU Framework on AMR Control 2020–2025, and AU Landmark Report on AMR.

Across the five countries, overall alignment with WHO GAP stewardship recommendations was moderate (53%). All NAPs reviewed had strong alignment with the GAP on items 1, 2 and 6 (national planning, limited access and stewardship programs), but poor alignment on Items 3 and 7 (quality-based market authorization and de-incentivizing inappropriate use). South Africa and Kenya recorded the highest alignment (61%), while Sudan had the lowest (44%) (Figure 2 & Table 1).

**Table 1.**
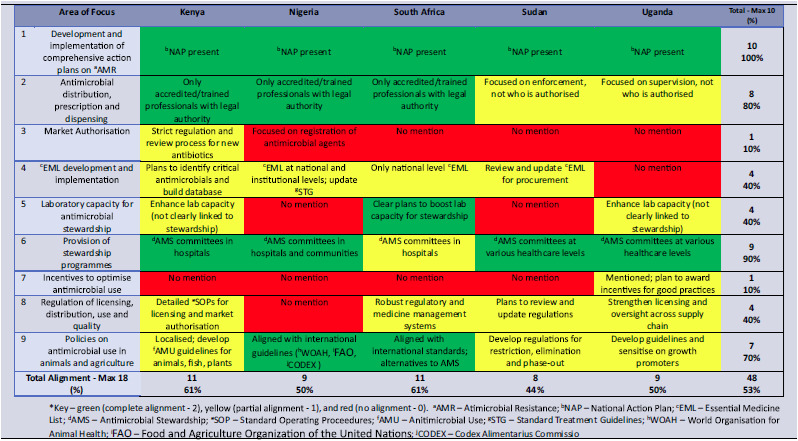
AMS alignment of selected NAPs with the GAP

#### Alignment with the AU framework for AMR control

The overall alignment with the AU Framework stewardship components was stronger, reflecting closer fit with regional priorities. Most NAPs demonstrated strong alignment (80%) with AMS items 1–3 (healthcare worker adherence in human and animal health, and regulation of substandard and falsified products). Alignment was poor on AMS item 4 (plant health). Nigeria, South Africa, and Uganda each recorded 75% with the AU Framework, while Kenya recorded 63% and Sudan 38% (Figure 2).

#### Alignment with the AU AMR landmark report

The countries average stewardship alignment with the AU AMR Landmark was 57%, with substantial variation between countries. Strong alignment was observed with AMS items 1, 3, and 6 of the AU Landmark Report (facility stewardship programs, in-service training, and access to quality antimicrobials and diagnostics), while item 4 on stewardship awareness campaigns showed the weakest alignment. South Africa achieved the highest alignment score (92%), while Sudan had the lowest (33%) (Figure 1 & Table 2).

**Table 2.**
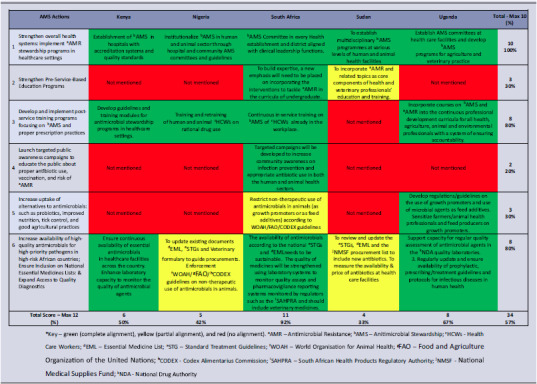
AMS alignment of selected NAPs with the AU AMR landmark report

## Discussion

This policy analysis highlights three cross-cutting stewardship challenges across Africa. First, African NAPs place strong emphasis on facility-based AMS interventions, particularly the establishment of stewardship committees, development of treatment guidelines, and training of healthcare workers within health facilities. While these measures are feasible and aligned with international recommendations, the NAPS placed comparatively limited attention to upstream market regulation and the economic drivers of antimicrobial misuse. Across the NAPs reviewed, weak alignment was observed for actions related to quality-based market authorization and the removal of incentives that encourage inappropriate antimicrobial use. This is notable in Africa where informal and poorly regulated antimicrobial markets are widespread, and access without prescription remains common.^7^ The focus on downstream stewardship within facilities, without parallel strengthening of regulatory and market controls, risks limiting the overall effectiveness of AMS strategies across African countries.

Secondly, stewardship efforts in the reviewed NAPs remain largely concentrated in human and animal health, with minimal integration of plant health and antimicrobial use in crop production. Although the AU Framework for AMR Control explicitly recommends extending stewardship to plant producers and promoting good agricultural practices, most NAPs showed little alignment with this component. References to antimicrobial use in agriculture were largely confined to livestock and aquaculture, while antimicrobial pesticides and their regulation were largely absent. This narrow sectoral focus undermines the implementation of a fully operational OH approach, particularly in African countries with large smallholder farming population and rapidly expanding agricultural input markets.^8^ This represents a critical gap in addressing environmental and agricultural pathways of AMR.

Lastly, public engagement and education are core pillars of the AMR response, yet AMS-focused public awareness and preservice training were underutilized across the NAPs examined. While most plans prioritized in-service training for healthcare professionals, fewer included provisions for integrating AMS into preservice curricula or for linking public awareness campaigns directly to responsible antimicrobial use. Where awareness activities were mentioned, they were often framed broadly around AMR rather than explicitly tied to stewardship behaviors among patients, caregivers, farmers, and community members. This emphasis on professional training alone risks reinforcing responsible supply without addressing demand-side drivers of misuse. Given their potential for long-term behavioral change and sustainability, the limited use of preservice education and AMS-focused public engagement represents a missed opportunity within current national strategies of African countries.^9^

This study forms part of the succession planning within the Ducit Blue Foundation’s One Health Pan-African AMR Internship/Mentorship Programme. It reflects broader efforts to strengthen policy analysis and evidence translation capacity among early-career public health professionals in Africa. Such capacity building is critical for succession planning in Africa, ensuring that tomorrow’s public health workforce have a good grasp of AMR governance, to support the development of effective NAPs, and ensure that AMS strategies are informed by locally generated evidence.

Note that NAPs reflect country sovereignty and context-specific priorities. Alignment with global and regional frameworks may not fully represent national policy intent.

## Data Availability

Data supporting this study are included within the article and/or supporting materials.

## References

[1] Naghavi M , Vollset SE , Ikuta KS , et al. Global burden of bacterial antimicrobial resistance 1990–2021: a systematic analysis with forecasts to 2050. Lancet 2024;404:1199–1226. 39299261 10.1016/S0140-6736(24)01867-1 PMC11718157

[2] Africa Centres for Disease Control and Prevention. African Union AMR landmark report. Africa CDC, Addis Ababa; 2024.

[3] World Health Organization. Global Action Plan On Antimicrobial Resistance. Geneva, WHO; 2015. 10.7196/samj.9644 26242647

[4] African Union. African Union framework for antimicrobial resistance control 2020–2025. AU, Addis Ababa; 2020.

[5] World Health Organization. WHO policy guidance on integrated antimicrobial stewardship activities. Geneva, WHO. 2021. Available from: https://www.who.int/teams/surveillance-prevention-control-AMR/national-action-plan-monitoring-evaluation/library-of-national-action-plans.

[6] Willemsen A , Reid S , Assefa Y. A review of national action plans on antimicrobial resistance: strengths and weaknesses. Antimicrob Resist Infect Control 2022;11:90. 35739564 10.1186/s13756-022-01130-x PMC9229779

[7] Gulumbe BH , Adesola RO. Revisiting the blind spot of substandard and fake drugs as drivers of antimicrobial resistance in LMICs. Ann Med Surg 2023;85:122–123. 10.1097/MS9.0000000000000113 PMC9949790 36845783

[8] Bosire LO. The trade in pesticides: a toxic double standard. The Elephant. 2024. Available from: https://www.theelephant.info/analysis/2024/01/17/the-trade-in-pesticides-a-toxic-double-standard/.

[9] Etukakpan A , Selçuk A , Meilianti S , Kusynová Z , Bajis D. Antimicrobial resistance and stewardship education: supporting the pharmaceutical workforce in AMR and AMS. Int Pharm Fed. 2023.

